# FEASIBILITY OF A TABLET DESIGNED FOR OLDER ADULTS TO FACILITATE TELEMEDICINE VISITS

**DOI:** 10.1093/geroni/igz038.3534

**Published:** 2019-11-08

**Authors:** Carla Perissinotto, Cai Zhang, Tim Oseau, Danielle Balik, Caroline Sou, Claire Burnight, Kerry Burnight

**Affiliations:** 1 University of California, San Francisco, San Francisco, California, United States; 2 Grandpad, Minnetonka, Minnesota, United States; 3 USC, Los Angeles, California, United States

## Abstract

Introduction: By incorporating age-friendly technology into the care of older adults with high utilization, providers can address complex care needs more efficiently and urgently. Age-friendly technology has the potential to alleviate loneliness that occur with homebound older adults and affect healthcare outcomes. Specific Aims: 1) To evaluate the feasibility of integrating age-friendly telemedicine in a homebased primary care setting using. 2) To evaluate the effects of engaging older adults with age-friendly technology to address loneliness. Methods: UCSF Geriatric Medicine provided GrandPads to 21 patients ages 60 to 94 over a one year period. All research participants were community residing, predominantly low income, and homebound. There was no charge to the patients for the GrandPad. Providers who used the GrandPad with their patients were queried on their experiences. Patient questionnaires included a telemedicine readiness survey, satisfaction and UCLA 3-item loneliness scale. GrandPad tablets are commercially available and underwent an evaluation with the IT Security Risk Assessments team. Results: 100% of providers who used grandpad felt was easy to use and efficient. 83% felt the platform was easier than other platforms. 83% of patients felt the grandpad helped them connect to their providers and 82.5% felt the grandpad was easy to use and 78% felt more connected as a result of using grandpad. Conclusion: Patients and providers are satisfied with an older adults friendly tablet and it can easily be implemented in health care settings. Grandpads may also help older adults feel more connected.

